# In-Person Schooling Amidst Children’s COVID-19 Vaccination: Exploring Parental Perceptions Just after Omicron Variant Announcement

**DOI:** 10.3390/vaccines10050768

**Published:** 2022-05-12

**Authors:** Fadi Aljamaan, Ali Alhaboob, Basema Saddik, Rolan Bassrawi, Rasha Assiri, Elshazaly Saeed, Khalid Alhasan, Shuliweeh Alenezi, Mohammed Alarabi, Abdulkarim Alrabiaah, Yazed Alkriadees, Nora Al-Saud, Badi Alenazi, Ali A. Rabaan, Rabih Halwani, Fahad AlZamil, Mazin Barry, Ziad A. Memish, Jaffar A. Al-Tawfiq, Mohamad-Hani Temsah

**Affiliations:** 1 College of Medicine, King Saud University, Riyadh 11362, Saudi Arabia; faljamaan@ksu.edu.sa (F.A.); drhbooob@gmail.com (A.A.); ehamed@ksu.edu.sa (E.S.); kalhasan@ksu.edu.sa (K.A.); salenizi@ksu.edu.sa (S.A.); malarabi@ksu.edu.sa (M.A.); alrabiaah@ksu.edu.sa (A.A.); yalkriadees@ksu.edu.sa (Y.A.); noura.f.f.alsaud@gmail.com (N.A.-S.); fzamil@ksu.edu.sa (F.A.); mbarry@ksu.edu.sa (M.B.); 2 Critical Care Department, King Saud University Medical City, King Saud University, Riyadh 11362, Saudi Arabia; 3 Pediatric Department, King Saud University Medical City, King Saud University, Riyadh 11362, Saudi Arabia; rkbassrawi@ksu.edu.sa; 4 Department of Family and Community Medicine, College of Medicine, University of Sharjah, Sharjah 27272, United Arab Emirates; bsaddik@sharjah.ac.ae; 5 Sharjah Institute of Medical Research, College of Medicine, University of Sharjah, Sharjah 27272, United Arab Emirates; rhalwani@sharjah.ac.ae; 6 Department of Basic Medical Sciences, College of Medicine, Princess Nourah bint Abdulrahman University, Riyadh 11564, Saudi Arabia; raassiri@pnu.edu.sa; 7 Prince Abdullah Bin Khaled Coeliac Disease Research Chair, Pediatric Department, King Saud University, Riyadh 11362, Saudi Arabia; 8 Division of Pediatric Kidney Transplant, Organ Transplant Center, King Faisal Specialist Hospital and Research Center, Riyadh 11211, Saudi Arabia; 9 Department of Psychiatry, College of Medicine, King Saud University Medical City, King Saud University, Riyadh 11362, Saudi Arabia; 10 Pediatric Department, Alyamamah Hospital, Riyadh 14222, Saudi Arabia; bqalenazi@moh.gov.sa; 11 Molecular Diagnostic Laboratory, Johns Hopkins Aramco Healthcare, Dhahran 31311, Saudi Arabia; arabaan@gmail.com; 12 College of Medicine, Alfaisal University, Riyadh 11533, Saudi Arabia; 13 Department of Public Health and Nutrition, The University of Haripur, Haripur 22610, Pakistan; 14 Department of Clinical Sciences, College of Medicine, University of Sharjah, Sharjah 27272, United Arab Emirates; 15 Division of Infectious Diseases, Department of Internal Medicine, King Saud University Medical City, Riyadh 11362, Saudi Arabia; 16 Division of Infectious Diseases, Faculty of Medicine, University of Ottawa, Ottawa, ON K1H 8M5, Canada; 17 Research and Innovation Center, King Saud Medical City, Ministry of Health & College of Medicine, Alfaisal University, Riyadh 11533, Saudi Arabia; zmemish@ksmc.med.sa; 18 Hubert Department of Global Health, Emory University, Atlanta, GA 30322, USA; 19 Specialty Internal Medicine and Quality Department, Johns Hopkins Aramco Healthcare, Dhahran 34465, Saudi Arabia; jaffar.tawfiq@jhah.com; 20 Infectious Disease Division, Department of Medicine, Indiana University School of Medicine, Indianapolis, IN 46202, USA; 21 Infectious Disease Division, Department of Medicine, Johns Hopkins University School of Medicine, Baltimore, MD 21218, USA

**Keywords:** COVID-19 pediatric vaccine, SARS-CoV-2 Omicron variant, Omicron parents’ perceptions, COVID-19 variants and schools

## Abstract

**Background**: The SARS-CoV-2 Omicron spread fast globally and became the predominant variant in many countries. Resumption of public regular life activities, including in-person schooling, presented parents with new sources of worry. Thus, it is important to study parental worry about the Omicron variant, willingness to vaccinate their children, and knowledge about school-based COVID-19 precautionary measures. **Methods:** A national, cross-sectional, pilot-validated online questionnaire targeting parents in the Kingdom of Saudi Arabia (KSA) was distributed between 31 December 2021, and 7 January 2022. The survey included sociodemographic, COVID-19 infection data, parental and children vaccination status, attitudes towards booster vaccine, parents’ Omicron-related perceptions and worries, and attitude towards in-person schooling. **Results**: A total of 1340 participants completed the survey, most (65.3%) of whom were mothers. Of the parents, 96.3% either received two or three doses of the COVID-19 vaccine. Only 32.1% of the parents were willing to vaccinate their young children (5–11 years of age). In relation to their children 12–18 years of age, 48% had already had them vaccinated, 31% were planning to vaccinate them, and 42.8% were willing to administer a booster dose. Only 16% were more worried about the Omicron variant compared to the Delta variant. Residents of western KSA were more worried about Omicron compared to Delta. Parents worried about the Omicron variant and male participants were significantly less aware of school-based COVID-19 precautionary measures. Parents with post-graduate degrees and those having more children were significantly more inclined to send their children to school even if COVID-19 outbreaks could occur in schools, while parents who were more worried about the Omicron variant and were more committed to infection prevention measures were significantly less inclined to do so. **Conclusions:** Overall, parents had lower worry levels about the Omicron variant compared to the Delta variant. They had a higher willingness to vaccinate their older children compared to the younger ones. In addition, our cohort of parents showed high willingness to send their children to schools and trusted the school-based preventative measures. These findings can inform policy makers when considering school related decisions during the current or future public health crises.

## 1. Introduction

In November 2021, 23 months after the emergence of the original SARS-CoV-2 variant in Wuhan, China, the SARS-CoV-2 Omicron variant, the most recent variant of concern (VOC), was initially reported from South Africa [1]. This occurred on the backdrop of more than 11.4 billion COVID-19 vaccine doses administered globally, which is the largest global rollout in the vaccine’s history. The available evidence suggested that the Omicron variant might be the most transmissible variant to date, with risk of infection to all age groups, including young children [2,3]. Therefore, it was anticipated that the Omicron wave could result in large numbers of cases including children, especially with the resumption of normal gathering activities [4]. In order to prevent this new variant’s spread, to increase vaccination uptake, and address public concerns of the new variant and their adherence to public health and social measures (PHSM), avoiding crowds, maintaining social distance and wearing masks in closed spaces was advocated [4].

The resumption of in-person schooling in most countries in this academic year is another international challenge facing authorities and parents with the emergence of this VOC; this decision was taken officially, especially with regard to the high vaccination rates and high globally-attained herd immunity from natural infections. The appearance of Omicron might change parents’ perceptions and expectations of the resumption of in-person schooling and is a practical challenge of their trust of schools’ COVID-19 preventive measures. 

Public perception to the Omicron variant is challenged from different tangents, first, effective therapies for severe COVID-19 cases remains a challenging medical issue, particularly with more emerging variants [5,6]. Another challenge is vaccination effectiveness against the new (VOC)s especially with the continuously evolving SARS-CoV-2 variants that require the most flexible and deployable mRNA vaccine platform [7,8]. The Omicron variant has substantial resistance to neutralization by infection- and vaccination-induced antibodies, highlighting the demands for research on the continuing discovery of broadly neutralizing antibodies [9].

Only a few months after its announcement, the Omicron variant was recognized as the most prevalent variant in most countries, owing to its high transmissibility rate [10,11]. The announcement and subsequent spread of Omicron coincided with some nations’ relaxation of public health and social measures (PHSM), including resumption of in-person schooling and the removing of strict masking mandates. The Kingdom of Saudi Arabia (KSA) resumed regular in-person school activities for intermediate and secondary schools in September 2021, while elementary schools resumed in January 2022. At the same time, the Ministry of Education (MOE) launched a bundle of school based precautionary measures for early detection and containment of any COVID-19 reported cases within the school premises to prevent the spread of the virus and avoid outbreaks inside schools. The acceptance rates of the public to COVID-19 vaccination had been variable. It was estimated that at the end of 2021, 50% of the World Health Organization (WHO) member states achieved a target of 40% immunization, and this rate is <10% in low-income countries [12]. Studies from KSA have shown an acceptance rate for COVID-19 vaccine among the general population between 40.7–71% [13,14]. The rate was 70% among healthcare workers in the KSA [14].

In the context of these developments, we conducted this survey in KSA to explore parents’ views about vaccinating their children against COVID-19, their worry level of the newly emergent Omicron variant of SARS-CoV-2, awareness about school-based prevention measures against COVID-19, and their perception of school attendance in case of a COVID-19 outbreak inside their children’s schools.

## 2. Method 

### 2.1. Data Collection

This cross-sectional survey among parents in KSA was conducted from 31 December 2021 to 7 January 2022. Participants were invited by convenience sampling through various social media platforms, including Twitter posts, WhatsApp groups and email lists. The questionnaire was distributed electronically through SurveyMonkey© and included questions about the worry level from COVID-19 variants, COVID-19 infection status, COVID-19 vaccination status, willingness to vaccinate children against COVID-19, and awareness about school based precautionary measures against COVID-19. The survey tool was adopted from our previously validated research on COVID-19 parental perceptions, with modifications related to the new Omicron variant [15,16,17,18,19]. The final version of the survey was approved by the research team for language accuracy and clarity.

### 2.2. Ethical Approval

Participants were informed of the purpose of the study and their voluntary participation was obtained by consent at the beginning of the survey. Ethical approval was obtained by the institutional review board of King Saud University (21/01139/IRB).

### 2.3. Statistical Analysis

The mean and standard deviation were used to describe continuous variables, while frequency and percentage were utilized for categorically measured variables. The Histogram and the Kolmogorov-Smirnov statistical tests of normality were used to assess the statistical normality assumption of metric variables and the Levene’s test was used to verify the statistical equality of variances for metric variables as well. The associations in the multivariate linear regression analysis were expressed as unstandardized beta coefficients with their associated 95% confidence intervals. The Statistical Package for the Social Sciences (SPSS) version 21.0 was used for the statistical data analysis. The Stand-Alone FACTOR program (release 10.09.01) was used for the parallel analysis and the tests of dimensionality of the measured questionnaire variables [20]. The statistical significance level was considered at 0.050. 

## 3. Results

A total of 1340 participating parents completed the survey. All of them were married and 65.3% were mothers. Of the respondents, 46.9% were 35–44 years of age, and 23% were 45–54 years of age. Most (79.6%) of the respondents were Saudi citizens. The majority (76.4%) had a university degree, while the rest were equally split between having high school education or less (12.3%) and having higher post-graduate degrees (Master’s and PhD degrees) (11.3%). In terms of monthly household income (MHI), the majority (62.2%) had an income greater than 10,000 SAR (2667 US$) per month (Table 1). 

21.8% of the respondents were unemployed/housewives or retired, 23% were healthcare workers, and 47% reported as employed. Geographically, 66% of the respondents were from the central region of the KSA, followed by the western region (15,1%). The mean household size was five (SD = 1.6), with an average number of three children (SD = 1.6). Nearly two thirds reported having a child aged 5–11 years, and 50% reported having a child aged 12–18 years. Only 9.9% reported having a child with a chronic mental or physical illness. 

Of the parents, 72.8% had never had COVID-19 and 25.7% had the infection but did not require hospitalization, and only 1.5% required hospitalization for COVID-19. On the other hand, 62.2% of the participants denied any of their family members had contracted COVID-19, but 35.4% reported that at least one family member had the disease without requiring hospitalization, while 1.8% had a family member who required general ward admission and 0.6% required intensive care admission for COVID-19.

Regarding parents’ perceived commitment to the COVID-19 precautionary measures, universal masking ranked as the highest (Mean = 4.26/5, SD = 1.10), followed by social distancing and avoiding crowds (Mean = 3.86/5, SD = 1.11). Avoiding handshaking was ranked the lowest in terms of commitment (Mean = 3.31/5, SD = 1.30).

Based on the reported COVID-19 vaccination status, 61.3% of the surveyed parents received the first two doses and 35% received a third booster dose, while 0.6% did not receive any dose due to unspecified reasons and 0.5% did not receive any dose due to medical reasons. Finally, 2.5% reported refusing to receive the vaccination due to their disbelief in COVID-19 vaccination. Moreover, 30.1% of the respondents reported regular compliance with annual flu vaccinations (Table 2).

### 3.1. Parental Worry from the Omicron Variant 

**Figure 1:**
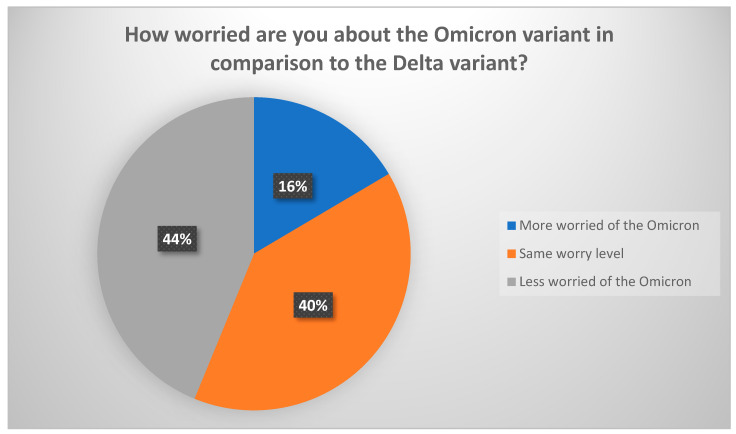
Parents’ Omicron worry level in comparison to the Delta variant.

**Figure 2:**
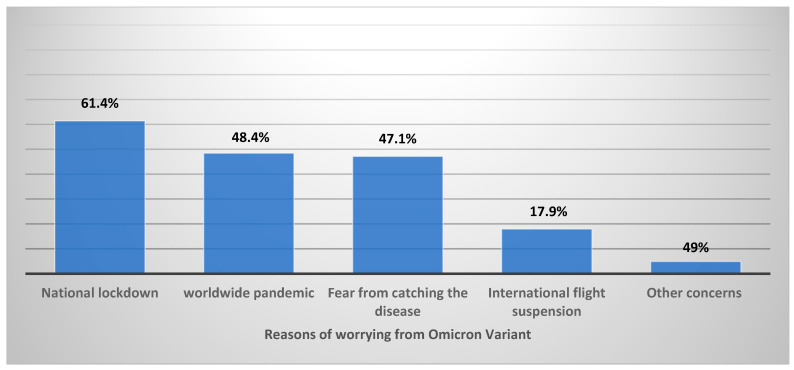
Parents’ reasons for worry in relation to the Omicron variant.

Reporting on their worry levels from the Omicron variant compared to the Delta variant, 40% of the surveyed parents were equally worried about both variants, while 44% were less worried about Omicron, and 16% were more worried about the Omicron variant (Figure 1). Parents reported that their top reason for worrying was fear of another national lockdown because of the expected surge of cases (61.4%), fear of another global pandemic (48.4%), and fear of contracting the disease (47.1%) (Figure 2).

### 3.2. Children Vaccination Status and Parents’ Precautionary Measures and Willingness to Vaccinate Their Children

**Figure 3:**
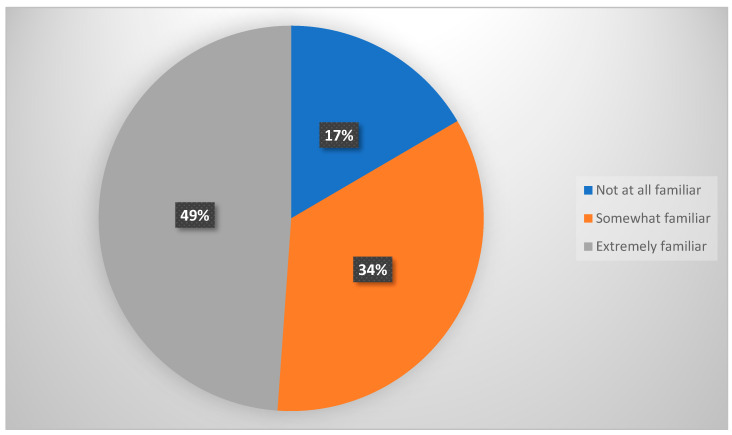
Parents’ awareness of schools’ precautionary measures against COVID-19.

In relation to parental levels of awareness of the precautionary measures against COVID-19 within schools, 17% of parents were not familiar at all, 34% were somewhat familiar, and 49% reported that they were very familiar with these measures (Figure 3). 24.8% of parents thought that their children should attend school in-person even if COVID-19 positive cases were reported in other classes.

**Figure 4:**
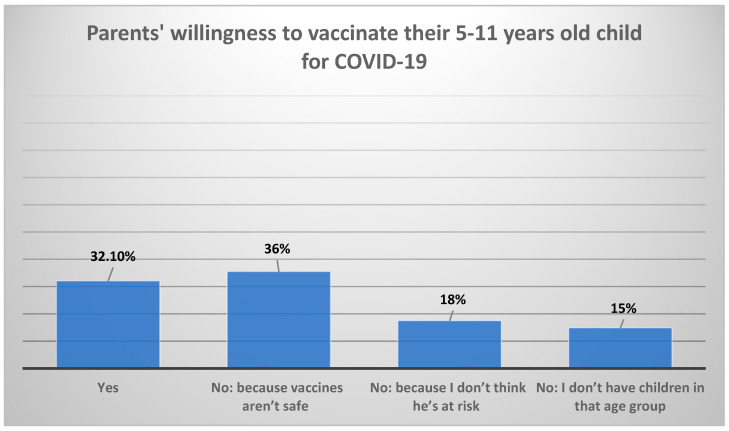
Parents’ willingness and reasons to avoid giving their children (5–11 years of age) the COVID-19 vaccine.

**Figure 5:**
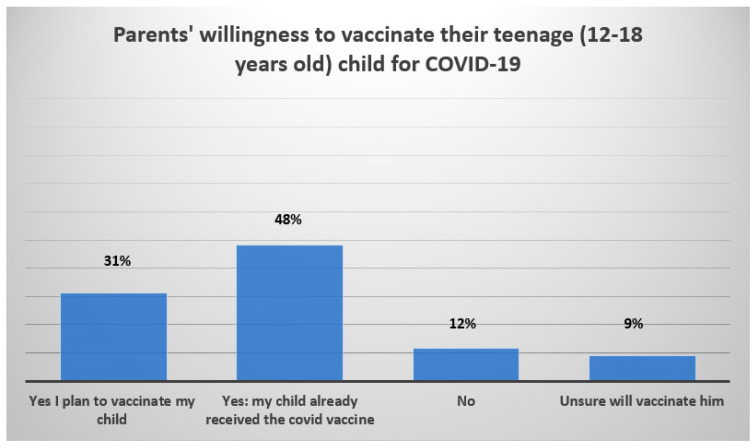
Parents’ reported COVID-19 vaccination status and willingness to vaccinate their teenaged children (12–18 years).

**Figure A1:**
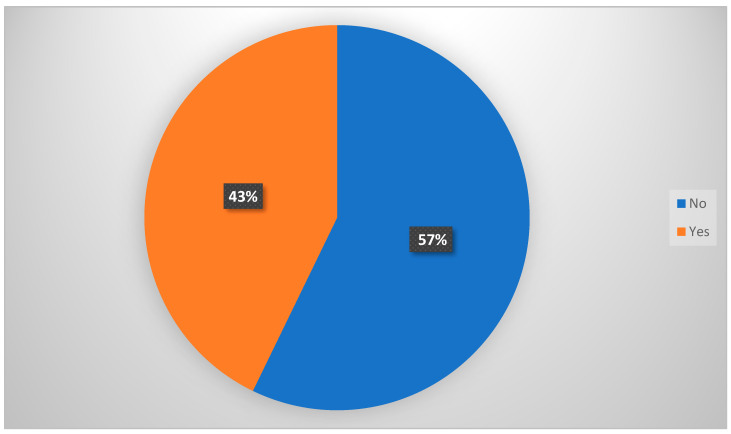
Parents’ willingness to give COVID-19 Booster vaccine to teenage (12–18 years) child.

**Figure A2:**
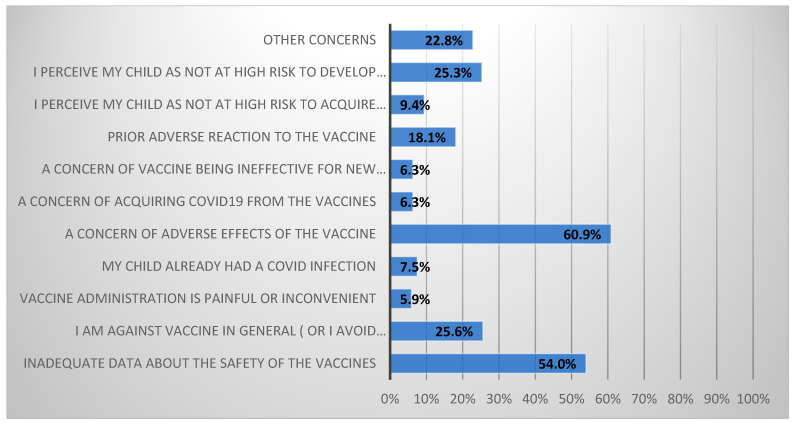
Parents’ reasoning for not giving their teenager children Booster COVID-19 vaccine.

**Figure A3:**
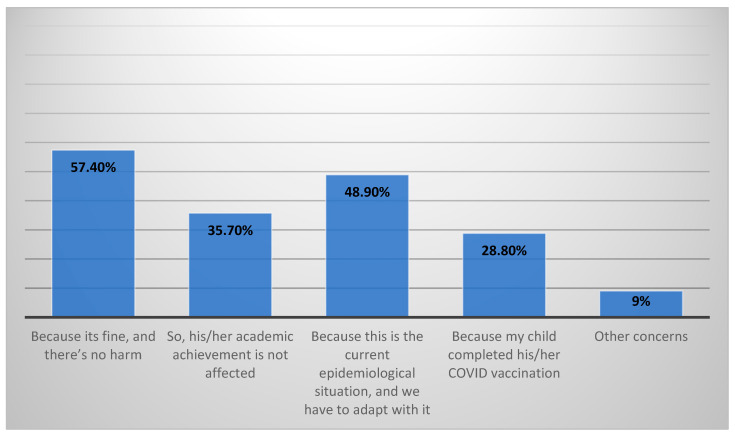
Parents’ reasoning of sending their teenager to school if COVID case reported in other classes.

**Figure A4:**
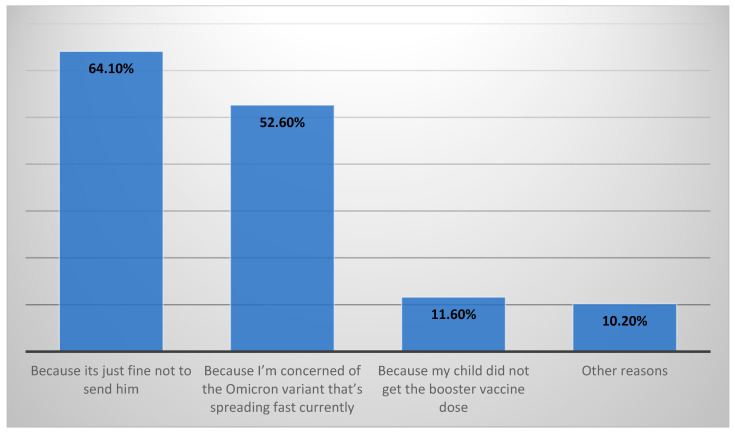
Parents’ reasoning of not sending their teenager child to school if COVID case reported in other classes.

Approximately 32% of parents surveyed agreed to vaccinate their children aged 5–11 years, 35.5% disagreed because they perceived vaccines as unsafe, 17.5% disagreed because they believed their children were not at risk, while 14.9% did not have children in that age group. Furthermore, 48% of them had already vaccinated their teenage children (aged 12–18 years), 31% were planning to vaccinate them, 12% denied vaccinating them, and 9% were hesitant. Regarding administration of the booster vaccine to teenage children, 42.8% of the parents indicated their willingness to administer it (Figure 4 and Figure 5). Appendix A Figure A1 and Figure A2 provide the description of parents’ willingness to administer the booster vaccine dose and a detailed description of the parents’ reasons for refusing a booster dose of COVID-19 for their teenaged children (12–18 years). Figure A3 and Figure A4 show the parental reasons for sending, or not, their teenager to school if COVID-19 case were reported in other classes. 

Table 3 shows a multivariable binary logistic regression analysis, which sheds light on the parental variables that were associated with higher worry levels about the Omicron variant compared to the Delta variant. Gender, age, willingness to vaccinate their children of any age, agreement with children’s school attendance, as well as parents’ or children’s COVID-19 immunization status did not correlate with any significance with higher worry levels from the Omicron variant. However, residence in the western Saudi Provinces was found to be significantly associated with more worry about Omicron (OR= 1.492, *p* = 0.048). The analysis also showed that higher numbers of children within a household were associated with lower worry levels concerning the Omicron variant (OR = 0.894, *p* = 0.041). Parents who have teenaged children (aged 12–18 years) were significantly more worried by Omicron (OR = 1.443, *p* = 0.037). In addition, parents who would not send their child to schools because of their concern of the high transmission rate of the Omicron variant were significantly more worried about the Omicron variant compared to the Delta variant (OR = 3.396, *p* < 0.001). On the other hand, parents who believed their children were not at a high risk of acquiring the disease were significantly less worried about Omicron (OR = 0.415, *p* = 0.002).

Table 4 shows the multivariable binary logistic regression analysis of the independent variables that were associated with parental odds of having low awareness of schools’ COVID-19 precautionary measures. Fathers had lower awareness when compared to mothers (OR = 1.689, *p* = 0.002). In addition, a family households’ monthly income was positively associated with their awareness level (OR for low awareness =0.856, *p* = 0.016). Saudi citizens had significantly lower awareness of schools’ COVID-19 precautionary measures when compared to expatriates (OR = 1.668, *p* = 0.023). Parents’ worry level concerning the Omicron variant was significantly associated with low awareness (OR = 1.696, *p* = 0.009). Moreover, parents who had children in the specified age groups (5–11 or 12–18 years of age) had significantly more awareness of schools’ COVID-19 precautionary measures (OR = 0.553, *p* = 0.001, OR = 0.512, *p* < 0.001 respectively). 

**Figure A5:**
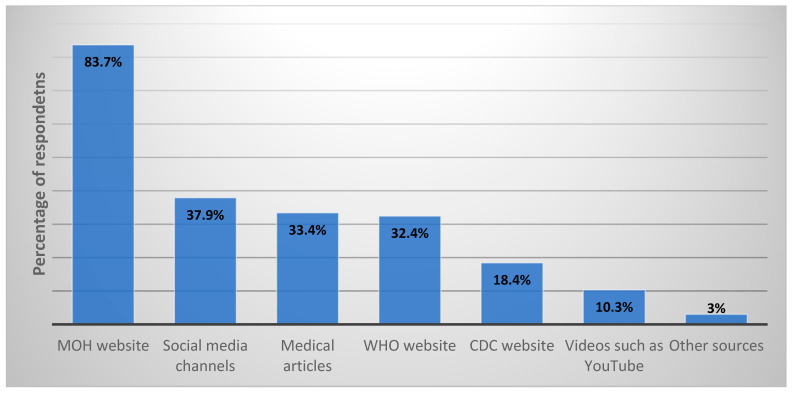
Participant’s sources of information for COVID-19 & vaccines.

Appendix A Figure A5 details parents’ different sources of information; parents who used the WHO website or as a source of information were found to be significantly less likely to have low awareness (OR = 0.560, *p* = 0.002). Parents who believed that their children should attend school even if there was an outbreak inside the school, and those who perceived the Omicron variant as a threat to school attendance owing to its high transmission rate were both significantly less likely to have low awareness of schools’ COVID-19 precautionary measures (OR = 0.440, *p* = 0.023, OR = 0.654, *p* = 0.027 respectively). 

**Figure A6:**
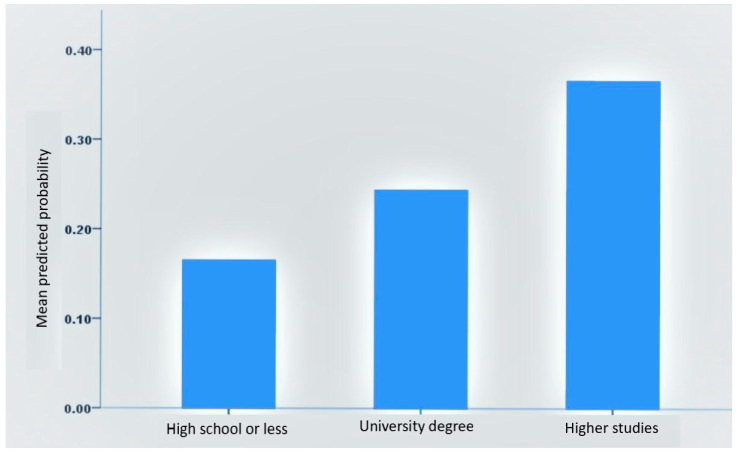
Mean predicted probability of parental agreement to send their children to school even if a COVID-19 outbreak happens inside the school in relation to their education level. Only parents with higher education level (Master or PhD) had significantly higher agreement (OR = 1.619, *p* = 0.014).

We analyzed our surveyed parents for their odds of agreement with attending school in person despite a COVID-19 outbreak inside the school using a multivariable binary logistic regression analysis. Table 5 details the variables significantly associated with agreeing to send children to school in the presence of a COVID-19 outbreak inside the school. Sex and age did not have a significant association according to this model. However, parents with higher educational levels (Higher levels of study such as a Master’s or PhD) were significantly more likely to agree to send their children to attend school in person even if an outbreak of COVID-19 was present (OR = 1.619, *p* = 0.014). (Appendix A Figure A6 shows the parental mean agreement predicted probability to send their children to school if COVID-19 outbreak burst inside the school in relation with their level of education).

Parents who were concerned about the Omicron variant compared to the Delta variant were significantly less inclined to send their children to school if there was a COVID-19 outbreak (OR = 0.322, *p* < 0.001). Parents of children in both specified age groups (5–11 or 12–18 years of age) were more likely to agree to send their children to school despite an ongoing outbreak (OR = 1.533, *p* = 0.013, OR = 1.701, *p* = 0.001, respectively). 

Additionally, parents willing to administer the booster vaccine dose to their teenage children (12–18 years of age) had similarly higher agreement (OR = 1.415, *p* = 0.013). Parents’ source of information for COVID-19 and its vaccines was significantly associated with this measure, as those who reported using the WHO website were significantly less likely to send their children to school in the presence of a COVID-19 outbreak (OR = 0.626, *p* = 0.004), while those who reported using the Centers for Disease Control and Prevention (CDC) website or medical journals were significantly more likely to agree to send their children to school (OR = 2.016, *p* < 0.001, OR = 1.669, *p* < 0.001 respectively). Higher commitment with COVID-19 infection prevention precautionary measures was associated with less agreement with attending schools in-person in the case of a COVID-19 outbreak (OR = 0.842, *p* = 0.013). 

## 4. Discussion 

This was a national survey targeting parents residing in the KSA investigating their worry levels and perceptions in relation to the Omicron variant and its relevance to vaccination and school attendance.

Adequate herd immunity in a community is needed to halt the ongoing spread of the COVID-19 pandemic. Different countries have been in a race to secure the needed supply of vaccines to reach that goal. But in addition to vaccine procurement challenges, vaccination efforts have been challenged by high levels of vaccine hesitancy and mistrust, therefore vaccination rates varied across different populations. Almost all of our cohort of participating parents received at least 2 doses of the COVID-19 vaccine, while only 3.2% did not receive any dose. That high rate of vaccine acceptance points to healthy parental attitudes toward vaccination, and studies from the adult Saudi population have shown COVID-19 vaccine acceptance rates ranging between 52–71% [13,21]. However, COVID-19 vaccination efforts in KSA for children have been affected by parental acceptance and hesitancy. In our study, among parents having younger children (5–11 years old in our cohort), only 32.1% were willing to vaccinate them, which is lower than other international surveys, where 69.2% of mothers (*n* = 11,800/17,054) indicated an intention to vaccinate their children [22]. 

Reasons for hesitancy in our cohort were similar to other studies, and included adverse effects, safety concerns, and their belief that this age group is not at risk or that their natural immunity is enough to prevent serious complications of the disease [23]. As there is variable COVID-19 vaccine acceptance among parents globally, vaccination campaigns should be tailored for each country and population in order to maximize vaccine uptake among children [22]. This could be balanced with the recent data showing children under five having lower risks of emergency department visits and hospitalization in the Omicron cohort (3.89% and 0.96%, respectively) as compared to the matched Delta cohort (21.01% and 2.65%, respectively) [24]. Regardless, the high number of patients seen during the Omicron surge can strain local health care systems [25]. Therefore, until more studies evaluate the virulence of this Omicron variant and its influence on public health, healthcare authorities need to maintain vigilance to ensure adequate vaccination uptake and the following of other prevention plans to avoid overwhelming numbers of COVID-19 infections, severe illness, or death [25,26].

Galanis et al. reviewed 44 studies that included 317,055 parents and found that 60.1% of parents intended to vaccinate their children against COVID-19. They also found that 22.9% of parents refused to vaccinate their children and 25.8% were unsure about it [27]. This lower tendency to accept the vaccine in younger children may be attributed to the higher parental perceived risk versus benefit in this age group. This is comparable to the literature that showed that the COVID-19 course is more severe in both infants and teenagers [28,29,30]. Among 57 studies with 21,549 patients that were included in the meta-analysis, Harwood et al. found that compared with children aged one to four years, infants had increased odds of admission to critical care and death. Also, the odds of death were increased in children aged 10–14 years and teenagers older than 14 years. In our study, there was a higher parental vaccination acceptance rate: 42.8% for teenage children 12–18 years in regard to receiving the COVID-19 vaccine and booster dose. 

Our cohort of parents also showed high commitment with universal face masking, which echoed similar findings from a study on the Saudi population showing 88.2% commitment. That study recorded 47.5% commitment to social gathering which is similar to the lower commitment to avoiding crowds in our cohort [31]. While our cohort had the lowest commitment of precautionary measures to hand shaking avoidance, another study showed very high commitment to this precautionary measure in the Saudi population, reporting an 82% compliance in avoiding it [32]. An Italian study of an elderly population aged over 65 years has shown above 95% belief in face masking, avoiding hand shaking and vaccination to prevent the disease, which is higher than the rates recorded in the Saudi population [33].

The Delta and Omicron variants are the ones most known by the public, mainly because the first was associated with higher morbidity and mortality while the latter was associated with higher contagiousness. Our cohort’s top reasons of with regard to the Omicron variant were running into a national lockdown following a surge of cases, followed by reigniting a worldwide pandemic which was almost equal to their fear of catching the disease. That highlights the huge and real impact of lockdowns globally, which was well demonstrated in many studies. Indeed, an estimated 2.6 billion people, one-third of the world’s population, were living under some form of lockdown or quarantine, arguably the world’s biggest psychological experiment, and an action to mitigate its toxic effects is highly needed [34]. Fear of catching the disease was found in 47.1% of responders, as this study took place in the first weeks of the new variant announcement and when no adequate information about disease severity was available. Factors associated with greater worry from Omicron compared to Delta can shed light on the correlates of increasing worry with the emergence of new variants of SARS-CoV-2. For example, parents who thought Omicron spreads faster were more worried about it compared to Delta. These findings are important since parents have had multiple stressful factors to deal with during the pandemic [35,36].

The potential higher transmissibility of Omicron has been discussed and was part of how parents perceived the risk to their children [37]. Residing in the western region of KSA was associated with higher worry form the Omicron variant, which may be related to the higher population density in this region of the country and their exposure to multi-nationalities related to pilgrimage to the holy cities of Makkah and Madinah in the western area of KSA [38]. Remarkably, having older children (12–18 years) was associated with increased worries of Omicron. A study has shown that increased child age was associated with worse health-related quality of life during the COVID-19 pandemic as assessed by both parents and children [39]. And given that it may be more difficult for parents to ensure that an older child is protected from infection by the new variant, this may explain the higher worries experienced by parents of teenagers. 

Moreover, our finding that parents with more children are less worried from Omicron indicates that they either underestimate the new threat or, possibly, cope better with the new variant. A previous report has shown that, although the difference was small, parents of more children were less likely to reach the cut-off score for anxiety during the COVID-19 pandemic [40]. Similarly, those who perceived their children’s risk of acquiring SARS-CoV-2 infection was low were less worried about the Omicron variant. These findings demonstrate the relationship between complacency about COVID-19 as a risk and parental worries and their intention to act in a protective manner [23,41].

The Saudi MOE was able to continue the educational process during the exceptional circumstances of the COVID-19 pandemic through e-learning and distance education systems. By the beginning of the academic year 2021/2022 the MOE resumed in a stepwise process, with in-person classroom group learning. It initially implemented the social distancing rules and later, by March 2022, reverted to the pre-pandemic regular system. The initial phase was accompanied by the implementation of preventive and precautionary measures recommended by health authorities. Our results showed that fathers had significantly lower awareness of those measures compared to mothers, a finding that contrasts with a Chinese study assessing parental awareness of COVID-19 protective measures for children, where fathers had more awareness [42]. Additionally, a US study showed that females were significantly less supportive of school-based COVID-19 risk mitigation measures [43]. Parents having children of school age (5–18 years) were more aware of the precautionary measures, which translated into a positive attitude towards sending their children to school even if a COVID-19 outbreak was present at the school. Such parental behavior is expected, given their interest in their children’s attending in-person school classes after two years of the pandemic and distance learning. This behavior also implied parents’ belief in the school precautionary measures applied by the authorities. Still, a minority of our cohort (24.8%) accepted their children attending the school in the case of a COVID-19 positive case reported in other classes; this could be due to fear of spread to the household, especially when considering the previously virulent variants. This fear and concern could lead to school absences as demonstrated by Lai et al. when they reported a substantial increase in the number of school absences in the UK in September 2020 when schools re-opened in England, as compared to 2019 [44].

Family income correlated positively with parental awareness of the schools’ preventive measures in our cohort. This might illustrate the healthy behavior of well supported families that care about their children and make sure to be updated about the school-based preventive measures while valuing their children’s education. This finding resembles reports from the US that show families with high annual income supporting School-Based COVID-19 risk mitigation measures and supporting in-person education [43]. 

Interestingly, parental worry levels from the Omicron variant was associated with a lower awareness level of schools’ COVID-19 preventive measures in our study. We believe this is an interesting finding that might be explained by worry fatigue. Schools had been closed for almost two years at the time of data collection, and there were ongoing debates on the value of their closure on ending the pandemic or decreasing its societal healthcare burden. At the same time, school closures had a negative impact on children’s mental well-being and academic performance. With all of this in the public’s mind, the announcement of the Omicron variant as a variant of concern might have caused worry fatigue for parents who were eager to be back to their regular lives including sending their children to in-person school activities [45]. Parents who were more aware of school-based precautionary measures and perceived Omicron as a threat to attend school were still in favor of attending school even if a COVID-19 outbreak happens at school. This might be explained by their belief that the Omicron variant was a risk but not to a degree that prevents school attendance if trustworthy precautionary measures were implemented. In our study, parents with higher commitment to infection prevention measures were less likely to send their children to school if a COVID-19 outbreak was present, and this is in contrast to a study showing good preparedness as a predictor of self-efficacy [46]. 

Furthermore, our finding that parents with higher educational levels and those willing to administer the booster vaccine dose to their teenage children were all in agreement in terms of sending their children to in-person school classes despite an ongoing COVID-19 outbreak echoed our previous finding that lower education was associated with parents favouring children staying home [47]. In a 2020 study from the US, planning to have children stay home was associated with fear of COVID-19, with no relation to race or ethnicity [47]. Another study showed that 56.5% of parents agreed with opening school but with racial differences in agreement [48,49].

### Study Limitations and Strengths

Although this study is subject to the usual limitations of cross-sectional studies, including sample size, technique, response bias, and potential recall biases, this research is nevertheless among the first to explore perceptions and worries among parents considering the SARS-CoV-2 Omicron variant amidst the resumption of the regular in person school activities. As the pandemic situation evolves, and variants are better understood and prepared for, parental experiences and perceptions are likely to change. Furthermore, as parental practices may differ from one country/locale to another, similar research in other countries is warranted to explore and address parental concerns with the Omicron global surge and the evolving pandemic stages. 

## 5. Conclusions

Our study demonstrated how parents residing in the KSA perceived Omicron and the precautionary measures against COVID-19 amidst the return to in person schooling in the country. Our results showed that parents are less worried about Omicron when compared to Delta, and that higher worry from Omicron was associated with certain factors such as fear of the high transmission rate of Omicron at schools and the age of children. Our results also demonstrate low parental commitment in terms of avoiding handshaking and avoiding gathering and crowds. In addition, parental willingness to vaccinate their 5–11-year-old children was very low compared to their willingness to vaccinate their teenaged children. Parents in our study were willing to send their children to in person school activities even if a COVID-19 outbreak was reported inside the school, and this was associated with higher parental educational level and higher awareness of precautionary measures inside schools. Finally, parents had high awareness of the school-based COVID-19 prevention measures, particularly mothers, which possibly reflects their belief and trust in the authorities and schools’ practices. These findings can inform policy makers when considering school-related decisions during the current or future public health crises.

## Figures and Tables

**Table 1:** Descriptive analysis of the parents’ sociodemographic characteristics.

Demographic Characteristics	Frequency (*n*)	Percentage (%)
**Sex**		
Female/mother	875	65.3
Male/father	465	34.7
**Age (Years)**		
25–34	267	19.9
35–44	628	46.9
45–54	308	23.0
55–64 or older	137	10.2
**Nationality**		
Saudi	1067	79.6
Non-Saudi	273	20.4
**Educational Level**		
High school or less	165	12.3
University Degree	1024	76.4
Higher studies (Master’s or PhD)	151	11.3
**Household Monthly Income**		
Prefer not to answer/unemployed	59	4.4
Less than 5000 SR	243	18.1
5000–10,000 SR	204	15.2
More than 10,000 SR	834	62.2
**Employment**		
Unemployed/Retired	292	21.8
Freelance	110	8.2
Healthcare worker	308	23.0
Employee	630	47.0
**Residence**		
Central region	885	66.0
Northern region	82	6.1
Eastern region	123	9.2
Southern region	48	3.6
Western region	202	15.1
Household Size *		5.0 (1.6)
Number of Children *		3.0 (1.6)
**Caring for Child Aged 5–11 Years**		
No	353	26.3
Yes	987	73.7
**Caring for Child Aged 12–18 Years**		
No	621	46.3
Yes	719	53.7
**Caring for Child with Chronic Physical/Mental Illness**		
No	1208	90.1
Yes	132	9.9
**Parent Had COVID-19**		
No	976	72.8
Yes, but did not require hospitalization	344	25.7
Yes, and required hospitalization	20	1.5
**Close Family Members Had COVID-19**		
No	833	62.2
Yes, but did not require hospitalization	475	35.4
Yes, and required hospitalization	24	1.8
Yes, and required hospitalization and ICU admission	8	0.6

* Mean (SD), SR: Saudi Riyals.

**Table 2:** Parents’ COVID-19 vaccination status and commitment to COVID-19 precautionary measures.

Variable	Frequency	Percentage
**Parent’s COVID-19 Vaccination Status**		
Yes: the primary two doses	821	61.3
Yes: with the third booster dose	469	35.0
No: due to a medical exception	7	0.5
No: I do not believe in the COVID-19 vaccine	35	2.6
Not received due to other causes	8	0.6
**Parent’s Seasonal Annual Flu Vaccination Status**		
Yes	403	30.1
No	937	69.9
**Family Commitment to Infection Prevention Measures**		
Universal masking in public places *		4.26 (1.10)
Social distancing and avoiding crowds *		3.86 (1.11)
Avoidance of handshaking *		3.31 (1.30)

* Mean (SD).

**Table 3:** Multivariable logistic regression analysis of parents’ odds of higher worry from the Omicron variant compared to the Delta variant.

Variable	(OR) *	95% C.I.		*p*-Value
		Lower	Upper	
**Gender**	1.026	0.733	1.436	0.883
Age	0.889	0.736	1.074	0.222
Parents who did not receive COVID-19 vaccine	0.314	0.073	1.354	0.120
Residence in western Saudi provinces	1.492	1.003	2.220	0.048
Number of children	0.894	0.803	0.996	0.041
Parents with teenage children (12–18 years of age)	1.443	1.022	2.036	0.037
Agreement with sending children to school despite the presence of COVID-19 cases at the school	0.584	0.306	1.113	0.102
Parents who perceive their children to not be at risk of acquiring COVID-19	0.415	0.236	0.729	0.002
Parents who perceive Omicron as a threat to school attendance due to its high transmission rate	3.396	2.370	4.867	<0.001

Dependent Variables (DV) = Greater worry from Omicron * Odds ratio.

**Table 4:** Multivariable Logistic Regression Analysis of parents’ odds of low awareness of schools’ COVID-19 precautionary measures.

Variable	(OR) *	95% C.I.		*p*-Value
		Lower	Upper	
Male	1.689	1.211	2.355	0.002
Age	0.856	0.709	1.035	0.108
Households’ monthly income >= 10,000 SR	0.817	0.693	0.963	0.016
Nationality (Saudi)	1.668	1.072	2.595	0.023
High worry level from Omicron	1.696	1.143	2.515	0.009
Number of children	0.917	0.815	1.032	0.149
Parents with young children (5–11 years of age)	0.553	0.383	0.797	0.001
Parents with teenage children (12–18 years of age)	0.512	0.355	0.739	<0.001
Parents willing to vaccinate their children (5–11 years of age)	0.693	0.449	1.068	0.097
Parents willing to vaccinate their children (12–18 years of age)	0.766	0.506	1.159	0.208
Mean perceived family commitment with infection prevention precautions	0.989	0.839	1.165	0.893
Source of information (WHO)	0.560	0.389	0.807	0.002
Source of information (Videos such as YouTube)	0.553	0.305	1.003	0.051
Parents who believe children should attend school even if an outbreak happens	0.440	0.216	0.895	0.023
Parents who perceive Omicron as a threat to school attendance due to high transmission rate	0.654	0.448	0.954	0.027

DV = Low awareness of school COVID Precautionary measures * Odds ratio.

**Table 5:** Multivariable Logistic Regression Analysis of parents’ odds of agreeing to send their children to school despite the presence of a COVID-19 outbreak in school.

Variable	(OR) *	95% C.I.		*p*-Value
		Lower	Upper	
Male	0.832	0.622	1.114	0.217
Age	1.001	0.840	1.193	0.990
Higher Educational Level	1.619	1.103	2.376	0.014
High worry level about Omicron compared to Delta	0.384	0.250	0.589	<0.001
Number of children	1.045	0.958	1.141	0.322
Parents with young children (5–11 years of age)	1.533	1.096	2.146	0.013
Parents with teenage children (12–18 years of age)	1.701	1.256	2.304	0.001
Parents with a child with mental/physical disability	0.721	0.459	1.134	0.157
Parents willing to administer the booster vaccine to their teenage children	1.415	1.075	1.863	0.013
Source of information (WHO)	0.626	0.457	0.857	0.004
Source of information (CDC)	2.016	1.417	2.869	<0.001
Source of information (medical articles)	1.669	1.260	2.212	<0.001
Parents’ commitment to the COVID-19 precautionary measures	0.842	0.736	0.965	0.013

DV = parental agreement with regard to attending school despite school COVID-19 outbreak * Odds ratio.

## Data Availability

Data is available upon reasonable request from the corresponding author by emailing to: mtemsah@ksu.edu.sa.

## Abbreviations

CDC	Centers for Disease Control and Prevention
COVID-19	Coronavirus disease 2019
MOE	Ministry of Education
SARS-CoV-2	Severe acute respiratory syndrome coronavirus 2
WHO	World Health Organization

## Appendix A

The most commonly reported reason for refusing a booster dose for their children (60.9%) was their belief the vaccines may have adverse effects followed by their perception of inadequate data on the safety of the vaccines (54%).

The majority of participants reported that their main source of information was the Saudi Ministry of Health website (MOH) (83.7%), followed by social media channels and medical articles (37.9%), the World Health Organization (WHO) website and the CDC website (32.4%, 33.4%, respectively), as well as other sources.
